# Ligand-Free Signaling of G-Protein-Coupled Receptors: Physiology, Pharmacology, and Genetics

**DOI:** 10.3390/molecules28176375

**Published:** 2023-08-31

**Authors:** Wolfgang Sadee

**Affiliations:** 1 Cancer Biology and Genetics, College of Medicine, The Ohio State University, Columbus, OH 43210, USA; wolfgang.sadee@gmail.com; 2 Bioengineering and Therapeutic Sciences, University of California, San Francisco, CA 94158, USA; 3 Aether Therapeutics Inc., Austin, TX 78756, USA

**Keywords:** GPCRs, ligand-free receptor signaling, inverse agonist, neutral antagonist, μ opioid receptor, opioid dependence, 6β-naltrexol, psychedelics, LSD, 5-HT2A, GHSR

## Abstract

G-protein-coupled receptors (GPCRs) are ubiquitous sensors and regulators of cellular functions. Each GPCR exists in complex aggregates with multiple resting and active conformations. Designed to detect weak stimuli, GPCRs can also activate spontaneously, resulting in basal ligand-free signaling. Agonists trigger a cascade of events leading to an activated agonist-receptor G-protein complex with high agonist affinity. However, the ensuing signaling process can further remodel the receptor complex to reduce agonist affinity, causing rapid ligand dissociation. The acutely activated ligand-free receptor can continue signaling, as proposed for rhodopsin and μ opioid receptors, resulting in robust receptor activation at low agonist occupancy with enhanced agonist potency. Continued receptor stimulation can further modify the receptor complex, regulating sustained ligand-free signaling—proposed to play a role in opioid dependence. Basal, acutely agonist-triggered, and sustained elevated ligand-free signaling could each have distinct functions, reflecting multi-state conformations of GPCRs. This review addresses basal and stimulus-activated ligand-free signaling, its regulation, genetic factors, and pharmacological implications, focusing on opioid and serotonin receptors, and the growth hormone secretagogue receptor (GHSR). The hypothesis is proposed that ligand-free signaling of 5-HT2A receptors mediate therapeutic effects of psychedelic drugs. Research avenues are suggested to close the gaps in our knowledge of ligand-free GPCR signaling.

## 1. Introduction

### Survey of the GPCR Superfamily

The superfamily of G-protein-coupled receptors (GPCRs) comprises ~800 gene encoding proteins with seven transmembrane domains [1], with over half dedicated to sensory functions, mediating olfaction (~400), taste (33), light perception (10), and pheromone signaling. Divided into six classes (A–F), the remaining 380 receptors have evolved as sensors detecting diverse and often minute stimuli, such as photons, pH, mechanical stress, metal ions, lipids, organic acids and bases, peptides and proteins, and more. These GPCRs account for a majority of clinically used drugs targets, across a broad range of applications [1,2]. Expressed in all cells, GPCRs regulate diverse physiological functions, maintain cellular homeostasis, and control cell growth. Similar to the range of activating stimuli, GPCRs also signal along multiple pathways [3,4,5,6], including heterotrimeric G proteins, arrestins, and more [7]. Upon stimulation, the canonical G-protein pathway progresses by exchange of GDP for GTP at the α subunit, followed by separate downstream signaling of active α subunits and βγ subunits, a multistep process with interconverting receptor conformations, modulated by allosteric interactions between ligands, components of the receptor complex, and the membrane environment [3,4,8,9]. Thus, GPCRs represent dynamic signaling complexes with multiple functional states, critical to understanding basal activity, ligand effects, and functional bias [5,10,11]. GPCRs can further exist in distinct oligomeric states, with higher order oligomers appearing in nanoclusters [4,12].

Because receptors are tuned to respond to minute triggers, one can expect spontaneous receptor activation to occur resulting in ligand-free signaling, common to most receptor types, including growth factor receptors that regulate cell proliferation [13]. Many GPCRs display detectable spontaneous signaling in ligand-free form. This review focuses on the following questions. Which GPCRs have been shown to exhibit ligand-free signaling? How does ligand-free signaling fit into existing GPCR signaling models? What are the physiological functions of ligand-free GPCR signaling? What are the implications of ligand-free signaling regarding pharmacological properties of drug ligands and for developing novel therapies? Lastly, what can we learn from genetic mutations about the role of ligand-free GPCR signaling?

## 2. GPCRs Displaying Documented Ligand-Free Signaling

Ligand-free signaling, also referred to as spontaneous, constitutive, or basal signaling, has been reported for diverse GPCRs [14,15,16,17,18,19,20,21], including virally encoded receptors supporting viral infections and cellular remodeling [22,23,24]. Several GPCRs feature masked or open tethered ligands. Protease-activated receptors (PAR1-4) are permanently activated by proteolytic cleavage of the extracellular N-terminus, which reveals a tethered N-terminal activating sequence [25,26]. Nevertheless, PARs can signal along various pathways through dimerization, activation by biased peptide ligands, allosteric interactions and cellular location including caveoli [25]. Whether the tethered ligand is permanently docked into the active site or rapidly dissociates and rebinds remains to be determined, which could enable an inverse agonist to block signaling. Adhesion GPCRs also display constitutive activity owing to a tethered peptide agonist [27]. In a similar fashion, the extracellular loop 2 of orphan receptor GPCR52 binds to the orthosteric site and maintains a high level of G-protein signaling but also features a separate pocket for ligand binding [28]. These examples highlight the broad spectrum of GPCR activation and ligand-free signaling.

Referred to as shape-shifters [16], GPCRs exist in multiple silent and active conformations signaling along diverse pathways. Ligands can interact preferentially with each of these states, leading to design of biased agonists and antagonists with favorable pharmacological properties [6,7,29]. Receptor states are stabilized by allosteric interactions with other membrane components such as cholesterol and proteins, generating receptor ensembles poised for ligand activation or already basally active in a ligand-free form. Conditions leading to ligand-free signaling include high receptor density [30] either by high expression or confinement in receptor clusters within cellular membrane compartments such as caveoli, shown with the neuropeptide Y2 receptor (Y_2_R) [31]. Also, GPCRs tend to dimerize or oligomerize with each other (homo-oligomers) or with other GPCR members (hetero-oligomers), forming higher order arrays that can account for biphasic and bell-shaped dose–response curves [3,4,25,30,31,32]. Activation of one receptor, either in ligand-free form or agonist stimulated, can allosterically interact with its neighbors, leading to a range of ligand-free signaling modes [3,4,30,33,34,35] Demonstrated for metabotropic glutamate receptors (mGluRs), a variable region within transmembrane helix 4 (TM4) contributes to homo- and heterodimerization and modulates orthosteric, allosteric, and ligand-free activation [36]. Similarly, a region at the interface of transmembrane domains of the obligatory heterodimer GABA_B2_ receptor confers cross-talk between the receptor monomers and controls its constitutive activity [17]. GHSR displays high basal activity, which conveys enhanced basal activity to other GPCRs it can bind to in a heterodimer complex [34,37,38].

While receptor internalization is typically viewed as a sequel to activating signaling, ligand-free internalization can occur spontaneously or after agonist dissociation [39,40]. If receptor internalization occurs after agonist dissociation, polar extracellular agonists such as serotonin or glutamate cannot readily enter the cell. Colocalization of ligands with internalized receptors indicates internalization with the bound agonist, as demonstrated with fluorescently labeled ligands for the parathyroid hormone receptor (PTHR) and the thyroid stimulating hormone receptor (TSHR) [41]. Alternatively, co-expression of cell membrane transporters of polar GPCR ligands can enable these ligands to reach internalized receptors (examples: OCT3 and β1AR1 [42]; EAAT3 and mGluR5 [43]).

Cellular compartmentation further affects GPCR signaling owing to distinct allosteric interactions, termed ‘cell location bias’ in signaling [41,42,43,44,45]. Sustained GPCR signaling after an initial stimulation can occur at the plasma membrane or in intracellular compartments. PTHR, TSHR, and mGluR5 signaling from endosomes, Golgi, and nuclear membranes is associated with prolonged cAMP or calcium signaling compared to those observed for plasma membrane signaling of these receptors, with intracellular mGluR5 signaling sufficient to regulate neuronal plasticity [41]. The activation of intracellular mGluR5 is sufficient to mediate long-term depression in hippocampal slices [43]. While the underlying mechanisms and consequences of prolonged signaling are poorly understood, one could hypothesize that enhanced ligand-free signaling plays a role; however, the role and regulation of ligand-free intracellular GPCR signaling have not yet been studied in any detail. Taken together, ligand-free signaling is a pervasive feature of GPCRs with broad physiological and pharmacological relevance.

## 3. Dynamics of GPCR Activation

The dynamics of receptor activation and interconverting ligand-bound and ligand-free receptor states are under intense study. The concept of multiple conformational and functional states of GPCRs [3,6,16,46] has led to increasingly complex receptor signaling models, with a focus on an active ternary agonist ligand-receptor G-protein complex [11,47] thought to transition between distinct conformations, as shown with the β-adrenergic receptor [48]. Orthosteric ligands typically bind to the canonical seven transmembrane helices (7TM) binding pocket, while allosteric components of the receptor aggregate stabilize specific receptor conformations and facilitate interconversions between them, activating switches between functional states [49]. These processes determine the signaling response [50] accounting for distinct effects of biased agonists and antagonists [3,4,6,29]. Powerful methods probing conformational states reveal dynamics and cellular location of signaling receptor aggregates [10,51,52,53,54]. Spatial confinement in nanodomains at the cell membrane or intracellular compartments confers additional signaling specificity [44,51,54].

**Figure 1:**
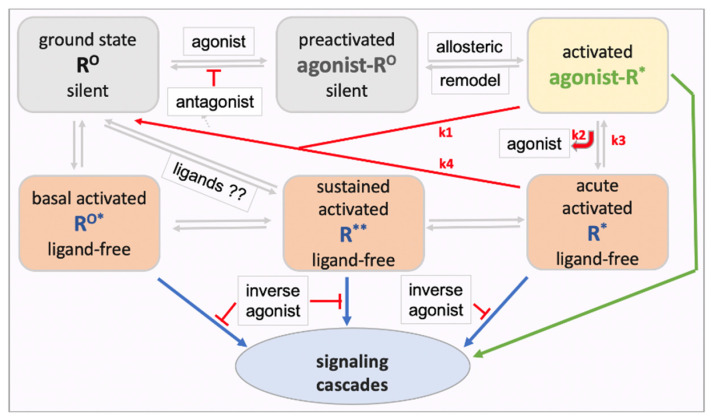
Proposed model of a GPCR with multiple interconverting conformational and functional states. The model includes both ligand-receptor (green) and ligand-free receptor (blue) signaling. Each receptor state could exist in varying complexes and subcellular locations with distinct signaling cascades. Both neutral antagonists and inverse agonists can block R^O^ activation in competition with agonists, whereas only inverse agonists can non-competitively block the ligand-free states R^O^*, R*, and R**. The extent to which acutely activated ligand-free R* accounts for overall signaling of an agonist depends on the rate constants k1, k2, and k3. The R** state is defined by altered ligand-free signaling after drug pretreatments and washout, or after physiological stimuli. The model has profound implications for potency and efficacy of agonists and antagonists, and it poses the hypothesis that ligands can accelerate interconversions between ligand-free signaling receptor states and the ground state R^O^.

Two hypotheses beyond the accepted basal ligand-free GPCR signaling could have general applicability and relevance for GPCRs: First, upon formation of the ternary agonist-receptor G-protein complex, activation of the G protein and downstream signaling processes could diminish agonist affinity, resulting in rapid agonist dissociation, while the ligand-free receptor complex continues to signal. Second, ongoing receptor signaling and other cellular processes regulate ligand-free signaling, thereby setting a new basal activity that can last for an extended time beyond the acute stimulus effect. The resulting receptor model illustrated in Figure 1 has been presented previously in similar form [55]. Upon engaging an agonist, the receptor ground state R^O^ adopts a preactivated conformation (ago-R^O^) followed by conversion to the accepted ternary ligand-receptor G-protein active-signaling complex (ago-R*). Ligand-free signaling can occur in three ways. First, many GPCRs spontaneously signal under normal physiological conditions, termed here ***basal ligand-free signaling*** (R^O^*). Second, ligand-free signaling can continue after agonist activation when the ligand dissociates from the receptor, potentially accounting for part of most of the signaling process with the agonist serving as a trigger—termed here ***acute activated ligand-free signaling*** (R*). Third, repeated agonist receptor activation or other physiological stimuli can result in sustained changes in receptor signaling long after the agonist is removed, termed here ***sustained ligand-free signaling*** (R**). Continued independent downstream signaling processes are not considered here. All receptor states are proposed to be interconvertible, modulated by ligands or physiological conditions. One can further assume that the receptor exists in more than one conformation or aggregate under each condition, resulting in various signaling cascades, not included in the model in Figure 1. Agonist-activated acute ligand-free signaling (R*) and sustained regulated ligand-free signaling (R** distinct from R^O^*) are not broadly recognized. Evidence supporting these proposed forms of ligand-free signaling will be discussed next, providing a guide for experiments to test the GPCR model in Figure 1.

## 4. Agonist and Antagonist Interactions with GPCRs Displaying Ligand-Free Signaling

### 4.1. Dissociation of Agonist Ligand from the Activated Receptor with Continuing Signaling

Agonists are proposed to stabilize the receptor in an ‘active’ conformation promoting G-protein coupling, which is thought to allosterically cause increased binding affinity for agonists and decreased antagonist affinity [46], thereby initiating signaling (activated ago-R*, Figure 1). This view is consistent with the observation that the four GPCRs tested (AR2A, GABA_B_, CB_1_R, and DRD2) are not pre-assembled with G proteins without agonist activation (except for basally active ligand-free CB_1_R) [50]. However, engaging the signaling pathway with subsequent dissociation of the G heterotrimer into G_α_ and G_βγ_ induces further remodeling of the active receptor complex [8,56], which could reduce agonist affinity. Experimental estimates of agonist residence time at the receptor, efficacy, and potency strongly depend on the experimental conditions, resulting in estimated values ranging over four orders of magnitude [57]. Both Na^+^ and GTP are needed for activating the G-protein signaling pathway [57,58], and both reduce receptor affinity in in vitro binding studies for agonists but not antagonists [58], facilitating agonist dissociation. To what extent the generated ligand-free R* contributes to the overall effect size and duration depends mainly on three rate constants: agonist-R* dissociation (k2) and reassociation (k3), and reversion of active ligand-free R* back to a silent ground state (k4) (Figure 1). Experimental evidence for this model comes from studies on rhodopsin [47] and opioid receptors [58]; however, direct evidence for a pervasive role of ligand-free R* among GPCRs is sparse.

The model depicted in Figure 1 further raises the question of whether the complete ternary ligand-receptor G-protein complex (ago-R*) and the acutely activated ligand-free R* are functionally distinct. The activation of GPCRs often results in a rapid first signal followed by prolonged signaling along distinct pathways, observed with TRH-R1, ADRB2, and NK2 receptors, suggesting an initial ‘induced fit’ followed by distinct subsequent events [59,60]. Also, morphine causes rapid Ca^++^ influx in μ opioid receptor (MOR) transfected HEK293 cells over the first 10 sec, followed by sustained intracellular Ca^++^ release [61]. GPCR activation commonly leads to clustering into nano-domains, a process that modulates signaling, as shown with the DRD2 receptor [54]. Pre-coupling with a non-canonical G protein followed by agonist activation resulted in enhanced agonist stimulated coupling to the canonical G protein [48], suggesting a priming effect that could occur during the ago-R* or the ligand-free R* states. In the absence of an agonist ligand, R* could be more susceptible to allosteric effects by other cellular components, facilitating a switch in receptor functions involving different G proteins and arrestins, and receptor forms that internalize and signal intracellularly. Biased agonists cause G-protein activation with or without subsequent internalization, as shown for morphine and DAMGO at the μ opioid receptor (MOR) [62]. Ligand-free R* could undergo further regulatory processes that establish long lasting signaling by ligand-free R**. These hypotheses require further testing.

### 4.2. Ligand-Free Signaling of Rhodopsin

The dim-light receptor rhodopsin (class A GPCR) has served as a premier experimental model, displaying structural features common to all GPCRs, such as multiple activation states and biased signaling [5,63,64]. Kept in an “off” state by a covalently bound inverse agonist, 11-cis retinal (11CR), rhodopsin is activated by photons via conversion of 11CR to the agonist all-trans retinal (ATR), activating the G protein transducin to yield the active state metarhodopsin II [47]. Metarhodopsin II is thought to continue signaling until the ATR-receptor link is hydrolyzed and ATR is released, causing conversion of MII into inactive opsin that can rebind 11CR.

Schafer et al. [47] have modified this rhodopsin model by including sustained ligand-free signaling after ATR dissociation, testing conformational dynamics with the use of time-resolved, fluorescence labeling experiments. They concluded that “an active-like, yet empty, receptor conformation can transiently persist after retinal release, followed by ATR rebinding, before the receptor ultimately collapses into an inactive conformation.” These observations support the receptor model with an agonist-R*—R* equilibrium (Figure 1). Their results further indicate that congenital blindness caused by constitutively active rhodopsin [64] might be treatable with inverse agonist drugs that disrupt the rebinding of ATR to active rhodopsin. Schafer et al. [47] further comment that continued ligand-free signaling after agonist dissociation could play an unappreciated role with other GPCRs as well.

### 4.3. Etorphine—An Ultra-Potent μ Opioid Receptor (MOR) Agonist

**Figure 2:**
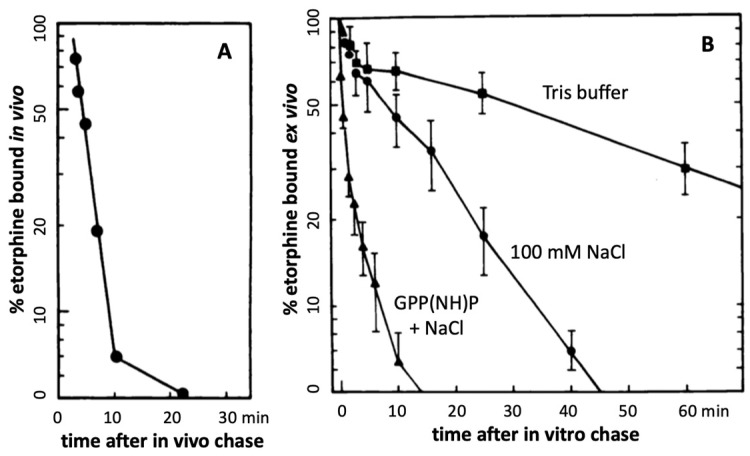
Rapid etorphine receptor dissociation in rat brain (adapted from Perry et al., 1982 [58]). A tracer dose of ^3^H-etorphine was injected s.c., labeling preferentially MOR sites, with ~50% of ^3^H-etorphine in the brain specifically bound at 17 min (time 0). At this time, a saturating chase dose of diprenorphine was injected, with sacrifice at the indicated time points, rapid brain homogenization, and analysis of bound ^3^H-etorphine (**A**). In panel (**B**), the animals are sacrificed at 17 min and saturating chase concentrations of etorphine are added in vitro (**B**). The in vitro chase experiment was performed in Tris buffer alone and with addition of 100 mM NaCl or 100 mM NaCl + GPP(NH)P (stable GTP analog) [58]. These results demonstrate rapid etorphine dissociation from MOR only under physiological conditions in vivo or immediately after sacrifice in the presence of NaCl + GPP(NH)P.

Further evidence in support of accelerated agonist-receptor dissociation after activation comes from experiments with the opioid agonist etorphine (antinociceptive EC50 ~0.0002 mg/kg in rats) ([58], and references therein). At this EC50 dose, the total amount of etorphine reaching the brain is far less than available MOR sites, leading to a MOR occupancy of only 2% [58]. Sufentanil causes antinociception in rats at a similar low fractional receptor occupancy [65]. A combination of three mechanisms can account for such extreme potencies. First, the agonist must have high affinity to the receptor, with association rates limited only by diffusion rate. A second mechanism key to extreme potency can result from continued ligand-free signaling after rapid agonist-receptor dissociation caused by reduced affinity to the active signaling receptor aggregate R*. Measured in vitro, etorphine’s dissociation half-life from MOR is ~30 min, partially accelerated by Na^+^ and GTP [58]. However, when injected in trace amounts in rats, followed by a chase with a saturating dose of an antagonist, the ^3^H-etorphine in vivo off rate from MOR in brain tissues is much shorter (t1/2 = 50 sec) (Figure 2A) [58], demonstrating greatly reduced affinity to the actively signaling MOR. One can reproduce this fast off-rate with in vivo labeling, followed by sacrifice, immediate brain tissue homogenization and incubation in vitro with a saturating etorphine chase dose in the presence of Na^+^ and GTP (Figure 2B) [58]. In the absence of NaCl and GTP, the dissociation is slow, matching that commonly observed in vitro (Figure 2B).

**Figure 3:**
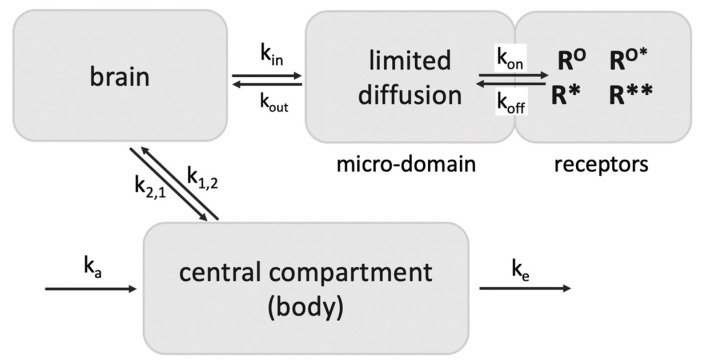
Retention of ligands at neurotransmitter receptors (GPCRs) in a receptor micro-domain in brain. This in vivo model with a diffusion-limited receptor micro-domain leads to retention of tracer doses at receptor sites (adapted from Perry et al., 1980 [66]), enabling selective receptor imaging in the brain. If k_out_ is smaller than k_on_, ligand rebinding occurs before diffusion away into brain tissues. For example, the opioid antagonist diprenorphine given in tracer amounts appears to rebind 7 times before exiting the micro-compartment, increasing its apparent t1/2 of dissociation from 18 min at MOR to ~2 h in vivo, longer than the elimination t1/2 from the circulation (45 min) in rats. As a result, >80% of a tracer dose in the brain is receptor bound [66]. Diprenorphine binds to all opioid receptors with high affinity.

These observations demonstrate rapid agonist-R* dissociation and indicate continued signaling by ligand-free R** in vivo. Third, assuming a diffusion barrier around the receptor sites (such as the synaptic cleft) and rapid rebinding, ligands at sub-saturating concentrations can rebind several times before diffusing away (modeled with a receptor micro-domain (Figure 3) [66]). Taken together, these processes can account for the extreme potency of etorphine and sufentanil, with the agonist serving as a repetitive trigger for generating active ligand-free MOR*. The same dynamics appear to apply to less potent MOR agonists including morphine. Both etorphine and morphine have antinociceptive EC50 values 2–3 orders of magnitude lower than the ID50 dose required to saturate 50% of ^3^H-etorphine sites in vivo (etorphine EC50 0.0001 mg/kg versus ID50 0.057 mg/kg, and morphine EC50 1 mg/kg versus ID50 180 mg/kg) [58,67]. Both ID50 values are far above lethal doses in rats. Similar receptor activation dynamics might also apply to the highly potent psychedelic lysergic acid diethylamide (LSD) acting at 5-HT2A receptors, and to a potent agonist of GHSR, both discussed further below.

### 4.4. Lysergic Acid Diethylamide (LSD)—An Ultra-Potent Agonist at 5-HT2A

LSD is one of the most potent 5-HT2A agonists, eliciting hallucinations with an oral dose at or above 0.025 mg in humans [68]. As discussed with etorphine, the amount of LSD reaching the brain is considerably below available 5-HT2A receptor sites assuming <1% of the dose reaches the brain. The acute effect peaks after ~3 h and lasts for 6–8 h, and up to 12 h after a high LSD dose (0.2 mg) [69]. The high 0.2 mg LSD dose results in peak blood levels of ~4 nM after 2 h, with 30% unbound LSD, leading to an estimated peak concentration of 0.7 nM in cerebrospinal fluid. This concentration is in the range of LSD’s in vitro binding affinity to 5-HT2A (Ki = 0.4–1.2) but below the EC50 for in vitro receptor stimulation (EC50 7 nM) [70,71]. Given the high level of 5-HT2A receptors in target tissues (~25 nM in rat brain frontal cortex [72]), LSD in vivo receptor occupancy is predicted to be low with LSD doses causing acute effects. After an early rapid distribution phase, the peripheral elimination half-life is 3–4 h, increasing to ~9 h in the terminal elimination phase, with LSD blood levels dropping far below the initial peak doses [70]. The drug level-effect relationship over time reveals negative hysteresis (the effect outlasting the blood level curve) [70,71]. Such extended effect has been interpreted to result from slow drug receptor binding equilibration, supported by the finding that LSD–5-HT2A dissociation studied in vitro is exceedingly slow (t1/2 ~3 h) [73,74]. However, such dissociation studies do not reproduce the in vivo conditions allowing active signaling, as demonstrated for etorphine (Figure 2). Assuming the LSD levels peak at <1 nM in the brain, only a small portion of 5-HT2A receptor sites can be occupied. Administration of labeled LSD to mice revealed early peak levels in the blood and brain and a relatively rapid removal from both compartments [75], incompatible with long selective LSD receptor retention in the brain. A parsimonious explanation for the potent pharmacological LSD effect is to invoke high affinity binding to 5-HT2A, followed by activation of receptor signaling, accelerated dissociation with continued ligand-free 5-HT2A* signaling, and multiple rebinding steps before diffusing away. Serving mainly as a trigger, LSD could generate robust acute effects at low receptor occupancy, as proposed for etorphine at MOR. While consistent with pharmacological observations, these predictions need to be directly tested experimentally. The implications of this hypothesis will be discussed in the section on psychedelic drugs.

## 5. Pharmacological Significance of Activated Ligand-Free Receptor Signaling

### 5.1. Involvement in Agonist Effects

Continuing signaling of ligand-free R* after agonist stimulation could be generally applicable to many GPCRs. If ligand-free R* accounts for a main portion of the agonist’s overall effect, agonists such as etorphine and LSD can reach extreme potencies as only a small fraction of the receptor population needs to be occupied. Partial agonists such as buprenorphine would need to occupy a larger portion of available receptor sites before sufficient activated agonist-R* and ligands-free R* is generated for analgesic effects [67]. The half-life of R* will determine effect duration when the agonist’s elimination half-life is shorter, resulting in counterclockwise hysteresis of concentration-effect–time curves (the effect outlasts the duration of agonist levels, for example, lisdexamfetamine [76]). While counterclockwise hysteresis (e.g., for morphine [77] and midazolam [78]) is modeled as a delay of equilibrium in a deep ‘receptor compartment’ [79] or slow receptor dissociation [80], continued ligand-free R* signaling provides a novel parsimonious mechanism, as discussed above with LSD. The extent and role of ligand-free R* signaling still has to be tested for most GPCRs. Given the large variety of agonist ligands, activation mechanisms are likely to differ between GPCRs.

### 5.2. Pharmacological Significance of Neutral Antagonism and Inverse Agonism

**Figure 4:**
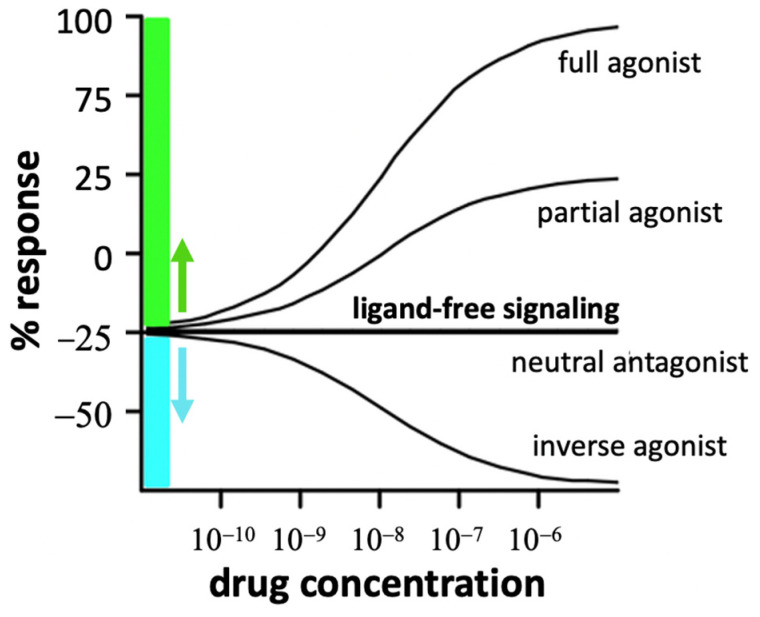
Ligand effects at a receptor displaying ligand-free signaling. Physiological changes and drug exposure can decrease or enhance basal ligand-free signaling (set at 0). Thus, a neutral antagonist can turn into a partial agonist or inverse agonist, depending on its efficacy at each of the receptor states (adapted from [29]).

Inverse agonists are defined by their ability to suppress ligand-free basal activated R^O^* signaling whereas neutral antagonists can bind but do not block R^O^* signaling (Figure 4). Efficacy levels at ligand-free R* and R** are mostly unknown. Ligand efficacies can vary between ligand-free signaling states of the same receptor depending on cellular conditions and pretreatments [81]. For example, naloxone and naltrexone are neutral antagonists at basal activated MOR^O^* but inverse agonists at MOR* or MOR** [82]. Similarly, pretreatment of DOR-transfected cells with an agonist and inverse agonist followed by washout changes ligand-free signaling and efficacies [83].

When ligand-free signaling contributes to pathophysiology, inverse agonists promise increased efficacy to correct pathophysiological conditions [84]. As both neutral antagonists and inverse agonists *competitively* block R^O^ activation by an agonist, one would expect equal antagonist potency with equal receptor affinity. However, in cases where ligand-free activated R* plays a main role in agonist stimulated signaling, potencies of neutral antagonists and inverse agonists diverge [29,55,85]. By definition, one would expect only the inverse agonist to block signaling of ligand-free R* in a non-competitive fashion, regardless of the agonist level present. Indeed, naloxone and naltrexone appear to be inverse agonists at ligand-free MOR* showing ~100-fold higher potency in blocking opioid antinociception and respiratory depression, or causing withdrawal in rhesus monkeys, than the neutral antagonist 6β-naltrexol even though receptor binding potencies are nearly equal [86]. The lower potency of 6β-naltrexol compared to naltrexone cannot be accounted for by the relatively slow access to the brain (as reported in rodents [87]), since we have found brain entry to be unimpeded in rhesus monkeys (unpublished data). In addition, naltrexone is highly potent (IC50 0.007 mg/kg) in blocking antinociception in mice caused by large doses of morphine (10–30 mg/kg) [88], acting dose-independently of morphine in a non-competitive fashion. Similarly, the high potency of naloxone and naltrexone in causing withdrawal symptoms, regardless of the opioid agonist load in the body, is consistent with a non-competitive mechanism involving inverse agonism at MOR* and MOR** [89]. Discrepancies between antagonist in vivo potency and in vitro receptor affinity of inverse agonists and neutral antagonists are predicted by the model in Figure 1 and need to be tested for other GPCRs as an indicator of prevalent ligand-free R* signaling.

Inverse agonists or neutral antagonists can be the drug of choice at ligand-free signaling GPCRs. For example, basal activity of β-adrenoceptors has led to the development of neutral and inverse agonists with distinct pharmacological effects [81]. For treatment of heart failure, a neutral β_1_-blocker such as carvedilol may lead to better survival outcomes than inverse agonist [21]. Modulation of 5-HT6R activity including its ligand-free signaling could serve to alleviate depression [90].

Antagonists can differ in their property as inverse agonist or neutral antagonist at the same GPCR as a function of the signaling pathway [4] or pretreatment with agonists or antagonists—demonstrated for opioid receptors [83,91] and serotonin receptors [92]. One can exploit these differences for designing biased agonists [6] and antagonists [4,30] that selectively activate or block one pathway over the other, not only through affinity differences between receptor conformations but also via inverse and neutral mechanisms.

Partial agonists are proposed to be less efficient than full agonists in generating agonist-R*, which can further convert into ligand-free R* (Figure 1). The partial agonist buprenorphine is used in opioid maintenance therapy but still can be diverted to illicit or recreational use, having sufficient agonist efficacy for non-medical use [93]. To avoid a diversion, a small amount of naloxone is co-formulated with buprenorphine in Suboxone^R^, in a ratio of 1:4 (e.g., 0.5 mg naloxone plus 2 mg buprenorphine) [94]. Upon oral administration, naloxone is largely metabolized in the liver during the first pass after absorption. However, when diverted for systemic use, this small amount of naloxone is sufficient to cause withdrawal even though buprenorphine’s in vitro receptor binding affinity at MOR is exceptionally high, with slow dissociation rates, suggesting that naloxone act non-competitively as inverse agonist at ligand-free MOR* in vivo. In animal studies, buprenorphine displays bell-shaped dose–response curves inhibiting its own action at high doses, above those administered to humans [95]. We had proposed the hypothesis that buprenorphine—with chemical features similar to the antagonist diprenorphine and an antagonist at κ opioid receptors—could rebind to the generated ligand-free MOR* at higher doses with reduced affinity, suppressing its signaling activity as an inverse agonist, and accounting for its bell-shaped dose–response curve [29]. Further studies are needed to understand the mechanism of action of buprenorphine and analogs, and applicability to other GPCRs.

## 6. Relevance of Ligand-Free R* Signaling to In Vivo Receptor Imaging Studies

For the interpretation of imaging studies of GPCRs with potent labeled ligands [96], the receptor model can serve to dissect agonist and antagonist binding profiles in vivo. For accurate positron emission tomography (PET) imaging of neurotransmitter receptors, for example serotonin receptors [97], a large portion of high affinity tracers present in the brain must reside at receptor sites to attain clear images. This phenomenon can result from selective retention in a receptor domain because of a diffusion boundary (for example the synaptic cleft) that slows ligand dispersal (Figure 3) [66]. As a result, the apparent dissociation rate at low receptor occupancy can be substantially higher than measured in vitro, resulting in selective long retention at receptor sites [66]. This retention is critical to in vivo receptor imaging in the brain.

Antagonists appear to bind to both silent and active receptors with high affinity, thereby labeling a large pool of receptor sites. On the other hand, agonist tracers label the same receptor with at least two distinct affinities thought to reflect low affinity binding to the uncoupled receptor and high affinity binding to the active agonist-receptor G-protein complex, as discussed with serotonin receptors [96,98]. Yet, an agonist tracer such as ^3^H-etorphine binds with high affinity to the receptor, but upon activation of signaling, appears to lose affinity at the agonist-R* state and dissociates more rapidly than shown in vitro [58]. Opioid agonists are considerably more potent in displacing a labeled agonist than antagonist tracers in vivo [65]. These dynamic differences between agonist and antagonist binding in vivo result in distinct displacement and saturation binding curves with increasing doses of agonists or antagonists [96]. As a result, saturation curves with opioid agonists tend to yield lower estimates of total receptor sites compared to those obtained with antagonists [96]. Similarly, agonists at the 5-HT2A receptor seem to label fewer binding sites than antagonists in transfected cells and are more potent in displacing agonist tracers than antagonist tracers at 5-HT2A [98], consistent with the receptor model in Figure 1. In addition, only agonist but not antagonist tracers can be detectably affected by competing levels of endogenous agonist ligands, demonstrated with serotonin in vivo load and 5-HT2A imaging with an agonist and antagonist tracer [97]. Distinct binding properties of agonists and antagonists are consistent with ligand-free R* signaling, yielding distinct in vivo labeling patterns as indicators of ligand-free R* signaling.

## 7. Physiological Significance of Sustained Ligand-Free Signaling (R** in Figure 1)

As ligand-free signaling of many GPCRs has physiological effects, one can assume that it is regulated by mechanisms similar to those applicable to agonist activated signaling, involving interactions with kinases, arrestins, and other regulatory factors. For example, MC4R displays basal ligand-free signaling and is involved in regulating appetite and metabolism. Its endogenous agonist α-melanocyte-stimulating hormone (α-MSH) causes anorexigenic effects, whereas its endogenous inverse agonist agouti-related peptide (AgRP) blocks MC4R ligand-free signaling with orexigenic effects [15], one example of endogenous inverse agonists regulating ligand-free receptor signaling. Receptor desensitization and internalization and subsequent degradation in lysosomes are thought to be the main mechanism by which permanently activated GPCRs can be switched off, as proposed for protease activated receptors (PARs) with an N-terminal tail that is cleaved to generate a tethered agonist peptide; yet, PARs continue to signal from endosomes with recruitment of β-arrestin [26]. Hence, PARs can display biased signaling even though the internal ligand is fixed, suggesting that the active receptor is responsive to allosteric factors [25] and potentially orthosteric ligands if the tethered agonist frequently dissociates and rebinds while the ligand-free PAR continues signaling, as demonstrated with rhodopsin [47]. If correct, inverse PAR agonists can be effective regulators of PAR signaling.

Subcellular location is critical to defining the signaling pathway [99,100]. For example, MOR and DOR residing in the Golgi apparatus couple to Gαi/o but not β-arrestin, unlike receptors in the plasma membrane [100]. GPCRs residing in intracellular organelles as well as newly internalized receptors can display ligand-free signaling that may proceed along pathways distinct for those occurring at the cell membrane. While receptor internalization has been linked to arrestin coupling, intracellular agonist-stimulated signaling can proceed via G-protein coupling, arrestin mediated signaling, and other mechanisms [100]. It remains to be determined for each GPCR whether receptor internalization occurs without a bound ligand, as shown with constitutively internalizing GPCRs [39,40]. When the agonist ligand is polar and does not penetrate the cell membrane (e.g., serotonin), intracellular agonist levels can be minimal unless gaining access via membrane transporters (e.g., glutamate and epinephrine) [42,43,44]. Whether intracellular GPCRs display a substantial degree of ligand-free signaling has yet to be studied broadly. Upon agonist stimulation, GPCRs tend to aggregate in cellular membrane compartments, assuming a punctate pattern when stained with fluorescent antibodies; such aggregation alone can activate ligand-free signaling by R** [31]. It is hence conceivable that repeated agonist stimulation results in sustained, enhanced ligand-free signaling in various cellular compartments (R** in Figure 1). However, while the activation status of GPCRs can be assessed with molecular probes [11,44,52,53,101,102], these methods typically address agonist activated pathways while little is known about intracellular ligand-free signaling and induced changes thereof for most GPCRs. If sustained ligand-free R** signaling were to play a physiological role, one must also address the question whether ligands, such as neutral antagonist, binding to R** can alter the equilibrium between receptor states, for example, resetting R** to the resting state R^O^ (Figure 1). While few studies address these issues, experiments with opioid and serotonin receptors reveal or suggest a role for regulated sustained R** signaling and ligand-mediated modulation of R**. Given the extraordinary diversity of GPCR activation and signaling mechanisms, it is difficult to project signaling models across all GPCRs. Rather, I will focus on opioid and serotonin receptors, and on the growth hormone secretagogue receptor GHSR, which displays high ligand-free signaling.

## 8. Opioid Dependence and Elevated Lasting Ligand-Free MOR** Signaling

The receptor model shown in Figure 1 has emerged largely from results obtained with molecular studies of MOR, combined with pharmacological data for interpreting in vivo ligand binding. Our work on receptor binding in live animals has revealed different properties than observed in vitro [29]. Repeated agonist stimulation causes sustained elevated ligand-free MOR** signaling, proposed to play a role in opioid analgesia and dependence [55,103,104,105]. The mechanisms of MOR** signaling are unknown but could involve β-arrestin-2 and c-Src signaling [106]. Agonist efficacy correlates with generated ligand-free MOR** signaling, as treatment of MOR transfected cells with the full agonist DAMGO caused a greater level of ligand-free MOR** signaling than the less efficacious morphine [107]. Elevated ligand-free MOR** signaling is revealed with inverse agonists such as naloxone and naltrexone that block R* and R** signaling and cause withdrawal symptoms long after agonist elimination from the body [89]. Basal ligand-free MOR^O^* differs from ligand-free R* and sustained R** because naltrexone and naloxone are neutral antagonists at basal ligand-free MOR^O^* but inverse agonists at both R* and elevated R** [82]. Delta and kappa opioid receptors (DOR and KOR) appear to have similar characteristics, with continued ligand-free signaling after agonist dissociation and altered states of sustained signaling as a function of agonist pretreatments [83,91,108]. Intracellular receptors could contribute to the cell’s ligand-free MOR signaling [45,53]. Dependence associated with elevated sustained MOR** could involve activation of tyrosine kinase signaling via arrestins [100] or via a calmodulin dependent pathway activating EGFR [109].

In contrast to naloxone, 6β-naltrexol does not cause withdrawal in an acute model of dependence (4 h after a single dose morphine) but blocks the effect of naloxone, indicating that endogenous opioids do not play a main role [89]. With chronic morphine pretreatment, 6β-naltrexol initially also causes withdrawal for several hours after the last morphine dose, but only with high 6β-naltrexol doses, while it no longer causes withdrawal symptoms 24 h after the last morphine dose, in contrast to naloxone [89]. These results could stem from distinct MOR** interactions of 6β-naltrexol compared to naloxone and naltrexone. Co-administration of guinea pigs with methadone and low 6β-naltrexol doses potently prevents the development of dependence in adult guinea pigs (IC50 ~0.01 mg/kg, measured 24 h after the last methadone dose with a naloxone challenge) at 6β-naltrexol doses far below its antinociceptive dose (IC50 ~1 mg/kg) [110]. 6β-Naltrexol also potently prevented morphine dependence in juvenile mice [111]. We hypothesize that 6β-naltrexol binds non-competitively to ligand-free MOR** and gradually accelerates conversion of elevated MOR** back to the ground state R^O^. As 6β-naltrexol is retained over long time periods in the brain (t1/2 ~5 h) after administration of small doses in mice and guinea pigs (unpublished data), low fractional MOR occupancy appears to be sufficient gradually to reverse the dependent state. Low-dose 6β-naltrexol also prevents neonatal withdrawal behavior in guinea pig pups born to dams exposed to methadone (IC50 ~0.025 mg/kg) [110].

The proposed reversal of ligand-free MOR signaling had been tested previously in mice with use of afferent nociceptors that express silent MOR sites [112]. Inflammatory signals alone (e.g., bradykinin release) are sufficient to generate ligand-free MOR** signaling—an example of physiological regulation of ligand-free signaling, which is acutely suppressed by naltrexone [112]. Naltrexone pretreatment alone generates ligand-free MOR signaling, detectable after naltrexone washout. In contrast, 6β-naltrexol reverses active ligand-free MOR** to the silent state and even blocks naltrexone’s activating effect [112]. This finding supports the hypothesis that ligands can affect interconversion between MOR** and silent MOR^O^, as depicted in Figure 1. The molecular properties of these proposed MOR states and interconversions remain to be experimentally tested.

The unique pharmacological properties of 6β-naltrexol led us to the hypothesis that 6β-naltrexol or its analogs could serve in the design of safer opioid analgesic co-formulations, weaning protocols, and prevention of neonatal abstinence syndrome [29,55,110]. How these concepts apply to other GPCRs remains to be determined.

## 9. Ligand-Free Signaling of Serotonin Receptor 5HT2A

### 9.1. Physiological and Pharmacological Relevance

A key neurotransmitter, serotonin has broad physiological functions interacting with serotonin receptors encoded by 12 genes. Among these, the 5-HT2A receptor serves as a common drug target in the treatment of schizophrenia and of depression indirectly by blocking serotonin transporters (SERT and SLC6A4). Drugs targeting 5-HT2A typically interact with multiple neurotransmitter receptors of high sequence homology, confounding a clear understanding of the underlying mechanisms responsible for clinical outcomes. In addition, 5-HT2A can form heterodimers with DRD2 [113] and the metabotropic mGlu2 receptor [114,115], conveying distinct functional properties in cells where they are co-expressed, involving three neurotransmitter pathways relevant to schizophrenia. Constitutive ligand-free activity of both 5-HT2A and 5-HT2C has been implicated in the etiology and treatment of affective disorders [116]. Inverse 5-HT2A agonists appear to have superior efficacy as antipsychotics compared to neutral antagonist [117,118], demonstrating the relevance of ligand-free signaling. Some antipsychotics such as pimavanserin are inverse agonists at the G_αi1_ pathway but neutral antagonists at the G_αq/11_ pathway. The G_αi1_ pathway is also considered the hallucinogenic effector pathway [92,119]. In view of the large in vivo potency differences between neutral antagonists and inverse agonists at MOR, the interpretation of these results remains unclear and raises several questions. Could 5-HT2A inverse agonists be merely more potent in vivo than neutral antagonist at the target signaling pathway? Does acutely activated 5-HT2A* play a role in response to agonists? Can ligands facilitate interconversion between 5-HT2A*^/^** states and the resting 5-HT2A^O^ state? Critical analysis of psychedelic drugs can help address some of these questions.

### 9.2. Psychedelic Drugs and 5-HT2A Signaling—Acute and Long-Term Effects

5-HT2A receptors are main targets for psychedelic drugs such as psilocybin, mescalin, and LSD [120]. Acting via heterodimeric receptor combinations, for example 5-HT2A-DRD2, psychedelics can further activate dopaminergic signaling [113]. Psychedelic assisted therapies have been tested in the treatment of intractable depression, post-traumatic stress disorder, addiction, chronic pain, and neurological disorders [121,122,123]—with the underlying mechanism remaining uncertain. Transient exposure to psychedelic drugs can result in long-lasting physiological and therapeutic effects [124] attributed to increased synaptogenesis and cortical dendritic spine size [122,125]. Low affinity 5-HT2A agonists such as dimethyltryptamine can also display psychedelic and long-lasting effects [126,127]. Empirical use of psychedelics is now followed by detailed basic and clinical studies, hampered by a patchwork of regulations at regional and central governmental levels [121].

Interactions between psychedelics with 5-HT2A receptors appear to be similar to those with opioid receptors. Structural analyses have determined the shape of ligand binding pockets and structures of the receptor with and without agonist ligands coupled to G_αi1_, G_q/11_, or β-arrestin-1 [74,92,119]. In addition, 5-HT2A signaling occurs intracellularly upon activation by lipophilic agonists including the endogenous serotonin metabolite dimethyltryptamine [102,128,129]. Serotonin cannot readily cross the plasma membrane and may not be the endogenous ligand for intracellular 5HT2A, while dimethyltryptamine is suggested to serve as internal ligand, an example of location bias in 5-HT2A signaling [102]. This hypothesis is consistent with the notion that serotonin does not internalize when bound to the receptor after activation at the cell membrane. However, dimethyltryptamine levels in the brain are rather low (~1 nM in pineal glands) [128] whereas binding affinity and agonist potency at 5-HT2A are moderate (in 5-HT2A-transfected cells: binding Ki 240 nM against ^3^H-ketanserine, EC50 76 nM in a Ca^++^ assay [120]). Therefore, endogenous dimethyltryptamine could occupy only a small fraction of 5-HT2A sites.

### 9.3. Long Lasting Effects of Psychedelics—Possible Role of Ligand-Free 5-HT2A Signaling

Single doses of psychedelics cause hallucinations over several hours, and extended mood changes for 1–2 days [70,124,130,131]. Single doses also lead to long lasting effects stimulating neuronal growth [102,125,131], exploited in various therapies, for example, in the treatment of depression and drug use disorders [123]. Conventional neuropsychiatric drugs also require days to weeks to begin working [122]. Ly et al. [125] assert that psilocybin-induced cortical neuron growth proceeds via initial 5-HT2A stimulation leading to TrkB activation and sustained neurite growth involving mTOR and AMPA receptor activation, which was heralded as a novel therapeutic target for the treatment of depression [102]. I propose the hypothesis that repetitive small incremental activation steps of 5-HT2A lead to cumulative increases in ligand-free 5-HT2A* and long lasting 5-HT2A** signaling, which can be maintained with low agonist receptor occupancy. Ligand-free signaling of neurotransmitter GPCRs has previously been proposed to provide tonic support for neuronal activity [132]. These hypotheses require more studies to be performed.

An alternative mechanism proposed to account for the antidepressant effects of psychedelics and antidepressants involves direct binding to TRKB receptors resulting in enhanced binding of neurotrophins to TRKB (e.g., BDNF) [133,134], the downstream target of 5-HT2A signaling [135]. However, the TRKB binding affinity of antidepressants appears to be too low to play a significant role in vivo. In contrast, LSD binds with a Kd of 0.6 nM to murine TrkB [133]. Moreover, a 0.1 mg/kg dose of LSD every 72 h in mice resulted in both a psychedelic-like response (head twitches) and antidepressant-like effects (blocking freezing response in cold water). Co-administration of a 5-HT2A antagonist blocked the psychedelic but not the antidepressant effect of LSD. Moliner et al. [133] concluded that LSD analogs could serve as novel antidepressants. However, it remains an open question as to whether direct activation of TRKB mediates or contributes to the antidepressant effects of LSD as the single dose of 0.1 mg/kg LSD used in mice is much higher than a hallucinogenic dose of LSD in humans (~0.001 mg/kg). This discrepancy is further highlighted by the lasting effects of psychedelic drug micro-dosing discussed below.

### 9.4. Micro-Dosing with Psychedelics—Attempts to Separate Hallucinogenic from Therapeutic Effects

Micro-dosing of psychedelics, at levels 5–10% of those causing hallucination, appears to suffice for attaining long-lasting effects as a potential therapy with broad applications [136,137]. The evidence is mostly empirical but gradually begins to meet rigorous standards. Clinical results raise the hypothesis that very low levels of psychedelics sufficiently activate 5-HT2A receptors, possibly involving intracellular 5-HT2A. Repeated receptor activation at low receptor occupancy could suffice to elevate 5-HT2A signaling in the form of ligand-free 5HT2A* or 5-HT2A**. Similarly, fluctuations of endogenous ligands such as dimethyltryptamine could regulate 5-HT2A receptor signaling. By this mechanism, dimethyltryptamine could indeed serve as a relevant endogenous 5-HT2A ligand [102], despite its low levels in the brain. Rather minute doses of psychedelics given over a prolonged time period could elicit the desired beneficial therapeutic effects without causing hallucinations, separating acute from chronic effects. This hypothesis has yet to be tested but could have relevance to drug development and treatment strategies.

## 10. Growth Hormone Secretagogue Receptor GHSR: High Ligand-Free Signaling

GHSR, and specifically its active splice-variant GHSR1a, is the receptor for the orexigenic peptides ghrelin and acetyl-ghrelin with a broad spectrum of physiological effects, such as stimulating growth hormone release, increasing hunger, modulating energy metabolism, and regulating immune function [138]. Expressed largely in the gastrointestinal tract, ghrelin mediates communication with the CNS via GHSR1a, highly expressed in the hypothalamus and pituitary, promoting the sensation of hunger [139] and regulating incentive value of artificial reward in substance abuse such as alcohol [140]. GHSR1a is of interest here because of its high level of ligand-free signaling [140,141]. Among several *GHSR* mutations, the Ala204Glu GSHR variant (rs121917883) selectively impairs ligand-free signaling [142,143]. The mutant receptor still responds to ghrelin activation [144], but in attenuated fashion, possibly in part because continued ligand-free signaling after agonist dissociation from the receptor is reduced. The Ala204Glu variant is associated with idiopathic short stature and growth hormone deficiency, demonstrating physiological relevance of ligand-free signaling. Internalized GHSR1a appears also to trigger signaling, possibly independent of ghrelin, by a pathway involving cAMP [138].

Signaling by GHSR1a is regulated on many levels, including by endogenous ligands, the potent agonist ghrelin/acyl-ghrelin and the inverse agonist LEAP2, both affecting eating behavior in opposing directions [145]. Similar to the role of agouti-related peptide as an inverse agonist of MCR4 [15], expression of an endogenous inverse agonist highlights the relevance of ligand-free GHSR1a signaling. Human plasma levels of ghrelin are approximately 170 pM, with the active acylated form of ghrelin still lower, fluctuating during the day and before taking a meal or during fasting [146]. The amount of acyl-ghrelin reaching hypothalamic-pituitary GHSR1a receptors likely occupies only a fraction of available receptors. Yet, acyl-ghrelin is extremely potent in further stimulation of GSHR1a activity, possibly causing enhanced ligand-free GHRS1a* signaling, as discussed for etorphine and LSD. The extracellular loop of GHSR1a may maintain enhanced ligand-free signaling functioning as an internal tethered ligand [15]. The hypothesis needs to be tested whether the agonist binds with a high affinity while active signaling causes agonist dissociation and enhanced ligand-free GHSR1a signaling, which is critical to understanding the extent and duration of action after dosing and guiding clinical trial design.

GHSR1a is a prolific partner, forming heterodimers with other GPCRs, including DRD1, DRD2, MC3R, and 5-HT2C, thereby allosterically modulating ligand-free signaling of its partner receptor [20,37,38]. Ligand-free signaling of GHSR itself is sufficient to enhance signaling of its partner [20]. Such interactions broaden the physiological influence of GRHS1a across multiple functions [38]. The regulation of ligand-free GHSR still remains to be studied in detail.

## 11. Genetic Variants That Affect Ligand-Free Signaling

Genetic variants can change ligand-free signaling of GPCRs in multiple ways. Enhanced or reduced expression will alter ligand-free signaling while enhanced receptor clustering could further promote ligand-free signaling. Changes in expression of receptor protein can result from genetic effects on transcription, RNA processing, translation, protein turnover and modifications, and cellular trafficking. Genetic variants altering protein structure and function can impact the degree of ligand-free signaling and efficacy of ligands including inverse agonists. A detailed study of genetic variants across populations has the potential to reveal insight on ligand-free signaling; yet, published studies fail to cover this spectrum of mechanisms, rather focusing on a limited set of functional assays related to basal ligand-free signaling (R^O^*).

The NaVa database lists natural GPCR variants [147], while the GPCRdb provides comprehensive information of all GPCRs [148], including native genetic variants and a majority of variants obtained by mutagenesis (65,536 missense variants) (https://gpcrdb.org, accessed on 1 March 2023). For example, the database lists 157, 102, and 105 missense variants for OPRM1, 5HT2A, and GHSR, respectively, including information on mutations that stabilize inactive/active states. GPCRs showing high ligand-free signaling—either natively or as a result of mutations—have been used for drug discovery [149] and ligand characterization distinguishing between neutral antagonists and inverse agonists [19]. Naturally occurring variants that alter ligand-free and agonist-stimulated GPCR signaling can reveal pathophysiological and pharmacological consequences [26,60,132,150]. For example, the basally active melanocortin MC4R harbors several loss-of-function mutants associated with obesity whereas gain-of-function mutants protect against obesity [151]. Constitutively active mutants have been detected for a number of GPCRs, including bitter taste receptors, chemokine receptor CXCR4 [18], adhesion GPCRs [152], angiotensin II type 1 receptor [153], thromboxane A2 receptor [154], and more.

Naturally occurring mutants of CXCR4 that promote enhanced signaling are linked to WHIM syndrome, a rare immunodeficiency disorder [18,155]. Ligand-free signaling appears to be enhanced by mutations at a dimerization interface that lead to monomerization to the active entity of CXCR4 [155]. Mutations can alter differential activation of various G proteins and G-protein-independent effects (biased agonism), dimerization-dependent effects, and interaction with allosteric modulators [156].

Loss or gain of function of endocrine receptors with functionally important ligand-free signaling has been associated with various endocrine disorders [156]. Inappropriate mutational activation of the thyroid stimulating hormone receptor (TSHR) beyond its native basal activity can lead to Grave’s disease [157]. Activating variants of rhodopsin cause blindness, a monogenetic disorder that might be treatable with inverse agonists [47,64,158]. As *GHSR* mutations have been shown also to result in stunted growth, GHSR is a drug target for the therapy of delayed growth in pediatric patients [159].

GPCRs play a key role in cancer by regulating tumor angiogenesis, immune evasion, metastasis, and drug resistance [160] and can serve as cancer drivers [161,162]. Large-scale surveys of the 1000 genomes projects [163] and the Cancer Genome Atlas [164], and other multi-omics approaches [165], have identified numerous GPCRs with aberrant regulation and mutations potentially driving cancers, but most remain to be studied in detail. Overexpression and activating oncogenic mutations have been reported for specific GPCRs in multiple studies [166,167,168,169]. For example, abnormal hedgehog pathway activation is a major driver of basal cell carcinomas and medulloblastoma [170], and drug resistance [171], offering drug targets in melanoma [172].

This brief synopsis highlights the importance of ligand-free signaling revealed by genetic variants.

## 12. Summary

This review offers new concepts and hypotheses about a pervasive role for ligand-free signaling of GPCRs and highlights gaps in our knowledge that require further studies. Three distinct receptor states are proposed for ligand-free GPCR signaling: basal/spontaneous signaling (R^O^*) observed for many GPCRs, acutely activated ligand-free signaling (R*), and sustained signaling of regulated ligand-free R** (Figure 1). Robust evidence supports substantial ligand-free R* and R** signaling for some receptors but is lacking for most GPCRs—a gap that needs to be filled in view of profound pharmacological and physiological consequences. Also proposed is the hypothesis that ligands binding to R* and R**, such as neutral antagonists and inverse agonists, can influence the equilibrium between these receptor states and the resting ground state R^O^ (Figure 1). 6β-Naltrexol is one such agent that prevents the developmenrt of opioid dependence possibly by reversing the elevated MOR** state back to the ground state R^O^ [55].

Several criteria can serve to test GPCRs for a substantive or dominant role of continued ligand-free R* signaling after agonist dissociation: agonists are effective at low receptor occupancy in vivo; agonist-receptor dissociation is accelerated in vivo during active signaling; agonist effects outlast receptor occupancy; agonists have a reduced ability to displace antagonist receptor binding in vivo; distinct pharmacological potencies between neutral antagonists and inverse agonists despite equal receptor affinities; and direct physicochemical demonstration of ligand-free signaling as demonstrated with rhodopsin [47]. While each criterion alone could have alternative explanations, taken together these criteria provide solid evidence.

The physiological relevance of regulated ligand-free receptor states (R**) is emphasized across several GPCRs, affording new avenues to manipulate ligand-free signaling. In particular, compounds changing lasting ligand-free signaling are potential drug candidates when given at doses below those affecting the acute pharmacological response. Such slow acting, low-dose regimen can result in novel therapies exploiting the physiological relevance of ligand-free GPCR signaling.

## 13. Patents

The following granted patents pertain to 6β-naltrexol and congeners: US8,883,817; 8,748,448; 9,061,024; and 10,925,870.

## Data Availability

Not applicable.

## References

[1] Alexander S.P.H., Christopoulos A., Davenport A.P., Kelly E., Mathie A., Peters J.A., Veale E.L., Armstrong J.F., Faccenda E., Harding S.D. (2019). CGTP Collaborators. The concise guide to pharmacology 2019/20: G protein-coupled receptors. Br. J. Pharmacol..

[2] Sriram K., Insel P.A. (2018). G protein-coupled receptors as targets for approved drugs: How many targets and how many drugs?. Mol. Pharmacol..

[3] Ferré S., Casadó V., Devi L.A., Filizola M., Jockers R., Lohse M.J., Milligan G., Pin J.P., Guitart X. (2014). G protein-coupled receptor oligomerization revisited: Functional and pharmacological perspectives. Pharmacol. Rev..

[4] Kurose H., Kim S.G. (2022). Pharmacology of antagonism of GPCR. Biol. Pharm. Bul..

[5] Perez D.M., Karnik S.S. (2005). Multiple signaling states of G-protein-coupled receptors. Pharmacol. Rev..

[6] Wingler L.M., Lefkowit Z.R.J. (2020). Conformational basis of G protein-coupled receptor signaling versatility. Trends Cell Biol..

[7] Eiger D.S., Smith J.S., Shi T., Stepniewski T.M., Tsai C.F., Honeycutt C., Boldizsar N., Gardner J., Nicora C.D., Moghieb A.M. (2023). Phosphorylation barcodes direct biased chemokine signaling at CXCR3. Cell Chem. Biol..

[8] Huang S.K., Pandey A., Tran D.P., Villanueva N.L., Kitao A., Sunahara R.K., Sljoka A., Prosser R.S. (2021). Delineating the conformational landscape of the adenosine A_2A_ receptor during G protein coupling. Cell.

[9] Lu M., Zhao W., Han S., Lin X., Xu T., Tan Q., Wang M., Yi C., Chu X., Yang W. (2022). Activation of the human chemokine receptor CX3CR1 regulated by cholesterol. Sci. Adv..

[10] Huang S.K., Prosser R.S. (2022). Dynamics and mechanistic underpinnings to pharmacology of class A GPCRs: An NMR perspective. Am. J. Physiol. Cell Physiol..

[11] Weis W.I., Kobilka B.K. (2018). The molecular basis of G protein-coupled receptor activation. Annu. Rev. Biochem..

[12] Dague E., Pons V., Roland A., Azaïs J.M., Arcucci S., Lachaize V., Velmont S., Trevisiol E., N’Guyen D., Sénard J.M. (2022). Atomic force microscopy-single-molecule force spectroscopy unveils GPCR cell surface architecture. Commun. Biol..

[13] Acevedo V.D., Ittmann M., Spencer D.M. (2009). Paths of FGFR-driven tumorigenesis. Cell Cycle.

[14] Bond R.A., Ijzerman A.P. (2006). Recent developments in constitutive receptor activity and inverse agonism, and their potential for GPCR drug discovery. Trends Pharmacol. Sci..

[15] Kleinau G., Heyder N.A., Tao Y.X., Scheerer P. (2020). Structural complexity and plasticity of signaling regulation at the melanocortin-4 receptor. Int. J. Mol. Sci..

[16] Liao C., May V., Li J. (2019). PAC1 Receptors: Shapeshifters in Motion. J. Mol. Neurosci..

[17] Liu L., Fan Z., Rovira X., Xue L., Roux S., Brabet I., Xin M., Pin J.P., Rondard P., Liu J. (2021). Allosteric ligands control the activation of a class C GPCR heterodimer by acting at the transmembrane interface. Elife.

[18] Luo J., De Pascali F., Richmond G.W., Khojah A.M., Benovic J.L. (2022). Characterization of a new WHIM syndrome mutant reveals mechanistic differences in regulation of the chemokine receptor CXCR4. J. Biol. Chem..

[19] Pydi S.P., Bhullar R.P., Chelikani P. (2014). Constitutive activity of bitter taste receptors (T2Rs). Adv. Pharmacol..

[20] Rediger A., Piechowski C.L., Yi C.X., Tarnow P., Strotmann R., Grüters A., Krude H., Schöneberg T., Tschöp M.H., Kleinau G. (2011). Mutually opposite signal modulation by hypothalamic heterodimerization of ghrelin and melanocortin-3 receptors. J. Biol. Chem..

[21] Velmurugan B.K., Baskaran R., Huang C.Y. (2019). Detailed insight on β-adrenoceptors as therapeutic targets. Biomed. Pharmacother..

[22] Couty J.P., Geshengorn M.C. (2005). G-protein-coupled receptors encoded by human herpesviruses. Trends Pharmacol. Sci..

[23] Davis-Poynter N., Farrell H.E. (2022). Constitutive signaling by the human cytomegalovirus G protein coupled receptor homologs US28 and UL33 enables trophoblast migration in vitro. Viruses.

[24] Rosenkilde M.M., Waldhoer M., Lüttichau H.R., Schwartz T.W. (2001). Virally encoded 7TM receptors. Oncogene.

[25] Canto I., Soh U.J., Trejo J. (2012). Allosteric modulation of protease-activated receptor signaling. Mini Rev. Med. Chem..

[26] Grimsey N., Lin H., Trejo J. (2014). Endosomal signaling by protease-activated receptors. Methods Enzymol..

[27] Wilde C., Fischer L., Lede V., Kirchberger J., Rothemund S., Schöneberg T., Liebscher I. (2016). The constitutive activity of the adhesion GPCR GPR114/ADGRG5 is mediated by its tethered agonist. FASEB J..

[28] Lin X., Li M., Wang N., Wu Y., Luo Z., Guo S., Han G.W., Li S., Yue Y., Wei X. (2020). Structural basis of ligand recognition and self-activation of orphan GPR52. Nature.

[29] Sadee W., Oberdick J., Wang Z. (2020). Biased opioid antagonists as modulators of opioid dependence: Opportunities to improve pain therapy and opioid use management. Molecules.

[30] Zhou B., Giraldo J. (2018). An operational model for GPCR homodimers and its application in the analysis of biased signaling. Drug Discov. Today.

[31] Sánchez M.F., Dietz M.S., Müller U., Weghuber J., Gatterdam K., Wieneke R., Heilemann M., Lanzerstorfer P., Tampé R. (2022). Dynamic in Situ Confinement Triggers Ligand-Free Neuropeptide Receptor Signaling. Nano Lett..

[32] Wang D., Sun X., Bohn L.M., Sadée W. (2005). Opioid receptor homo- and heterodimerization in living cells by quantitative bioluminescence resonance energy transfer. Mol. Pharmacol..

[33] Manglik A., Kobilka B.K., Steyaert J. (2017). Nanobodies to study G protein-coupled receptor structure and function. Annu. Rev. Pharmacol. Toxicol..

[34] Müller A., Berkmann J.C., Scheerer P., Biebermann H., Kleinau G. (2016). Insights into basal signaling regulation, oligomerization, and structural organization of the human G-protein coupled receptor 83. PLoS ONE.

[35] Mukherjee R.S., McBride E.W., Beinborn M., Dunlap K., Kopin A.S. (2006). Point mutations in either subunit of the GABAB receptor confer constitutive activity to the heterodimer. Mol. Pharmacol..

[36] Thibado J.K., Tano J.Y., Lee J., Salas-Estrada L., Provasi D., Strauss A., Marcelo Lamim Ribeiro J., Xiang G., Broichhagen J., Filizola M. (2021). Differences in interactions between transmembrane domains tune the activation of metabotropic glutamate receptors. Elife.

[37] Kern A., Grande C., Smith R.G. (2014). apo-Ghrelin receptor (apo-GHSR1a) regulates dopamine signaling in the brain. Front. Endocrinol..

[38] Wellman M., Abizaid A. (2015). Growth hormone secretagogue receptor dimers: A new pharmacological target. eNeuro.

[39] Jacobsen S.E., Ammendrup-Johnsen I., Jansen A.M., Gether U., Madsen K.L., Bräuner-Osborne H. (2017). The GPRC6A receptor displays constitutive internalization and sorting to the slow recycling pathway. J. Biol. Chem..

[40] Segredo V., Burford N.T., Lameh J., Sadée W. (1997). A constitutively internalizing and recycling mutant of the mu-opioid receptor. J. Neurochem..

[41] Crilly S.E., Puthenveedu M.A. (2021). Compartmentalized GPCR Signaling from Intracellular Membranes. J. Membr. Biol..

[42] Nash C.A., Wei W., Irannejad R., Smrcka A.V. (2019). Golgi localized β1-adrenergic receptors stimulate Golgi PI4P hydrolysis by PLCε to regulate cardiac hypertrophy. Elife.

[43] Purgert C.A., Izumi Y., Jong Y.J., Kumar V., Zorumski C.F., O’Malley K.L. (2014). Intracellular mGluR5 can mediate synaptic plasticity in the hippocampus. J. Neurosci..

[44] Irannejad R., Pessino V., Mika D., Huang B., Wedegaertner P.B., Conti M., von Zastrow M. (2017). Functional selectivity of GPCR-directed drug action through location bias. Nat. Chem. Biol..

[45] Stoeber M., Jullié D., Lobingier B.T., Laeremans T., Steyaert J., Schiller P.W., Manglik A., von Zastrow M.A. (2018). Genetically encoded biosensor reveals location bias of opioid drug action. Neuron.

[46] Liao C., May V., Li J., Ma N., Nivedha A.K., Vaidehi N. (2021). Allosteric communication regulates ligand-specific GPCR activity. FEBS J..

[47] Schafer C.T., Fay J.F., Janz J.M., Farrens D.L. (2016). Decay of an active GPCR: Conformational dynamics govern agonist rebinding and persistence of an active, yet empty, receptor state. Proc. Natl. Acad. Sci. USA.

[48] Culhane K.J., Gupte T.M., Madhugiri I., Gadgil C.J., Sivaramakrishnan S. (2022). Kinetic model of GPCR-G protein interactions reveals allokairic modulation of signaling. Nat. Commun..

[49] Unal H., Karnik S.S. (2012). Domain coupling in GPCRs: The engine for induced conformational changes. Trends Pharm. Sci..

[50] Bondar A., Lazar J. (2017). The G protein G_i1_ exhibits basal coupling but not preassembly with G protein-coupled receptors. J. Biol. Chem..

[51] Anton S.E., Kayser C., Maiellaro I., Nemec K., Möller J., Koschinski A., Zaccolo M., Annibale P., Falcke M., Lohse M.J. (2022). Receptor-associated independent cAMP nanodomains mediate spatiotemporal specificity of GPCR signaling. Cell.

[52] Elgeti M., Hubbell W.L. (2021). DEER analysis of GPCR conformational heterogeneity. Biomolecules.

[53] Irannejad R., Tomshine J.C., Tomshine J.R., Chevalier M., Mahoney J.P., Steyaert J., Rasmussen S.G., Sunahara R.K., El-Samad H., Huang B. (2013). Conformational biosensors reveal GPCR signalling from endosomes. Nature.

[54] Kovtun O., Torres R., Bellocchio L.G., Rosenthal S.J. (2021). Membrane nanoscopic organization of D2L dopamine receptor probed by quantum dot tracking. Membranes.

[55] Sadee W., McKew J.C. (2022). Ligand-Free Signaling of G-Protein-Coupled Receptors: Relevance to μ Opioid Receptors in Analgesia and Addiction. Molecules.

[56] Hilger D. (2021). The role of structural dynamics in GPCR-mediated signaling. FEBS J..

[57] Rinken A., Veiksina S., Kopanchuk S. (2016). Dynamics of ligand binding to GPCR: Residence time of melanocortins and its modulation. Pharmacol. Res..

[58] Perry D.C., Rosenbaum J.S., Kurowski M., Sadée W. (1982). ^3^H-Etorphine Receptor Binding In Vivo: Small Fractional Occupancy Elicits Analgesia. Mol. Pharmacol..

[59] Engel S., Gershengorn M.C. (2007). Thyrotropin-releasing hormone and its receptors—A hypothesis for binding and receptor activation. Pharmacol. Ther..

[60] Kleinau G., Jaeschke H., Mueller S., Worth C.L., Paschke R., Krause G. (2008). Molecular and structural effects of inverse agonistic mutations on signaling of the thyrotropin receptor—A basally active *GPCR*. Cell Mol. Life Sci..

[61] Quillan J.M., Carlson K.W., Song C., Wang D., Sadée W. (2002). Differential effects of mu-opioid receptor ligands on Ca(2+) signaling. J. Pharmacol. Exp. Ther..

[62] Arden J.R., Segredo V., Wang Z., Lameh J., Sadée W. (1995). Phosphorylation and agonist-specific intracellular trafficking of an epitope-tagged mu-opioid receptor expressed in HEK 293 cells. J. Neurochem..

[63] Park J.H., Scheerer P., Hofmann K.P., Choe H.W., Ernst O.P. (2008). Crystal structure of the ligand-free G-protein-coupled receptor opsin. Nature.

[64] Tsukamoto H., Farrens D.L. (2013). A constitutively activating mutation alters the dynamics and energetics of a key conformational change in a ligand-free G protein-coupled receptor. J. Biol. Chem..

[65] Rosenbaum J.S., Holford N.H.G., Richard M.L., Aman R.A., Sadee W. (1984). Discrimination of three types of opioid binding sites in rat brain in vivo. Mol. Pharmacol..

[66] Perry D.C., Mullis K.B., Oie S., Sadée W. (1980). Opiate Antagonist Receptor Binding In Vivo: Evidence for a New Receptor Binding Model. Brain Res..

[67] Rosenbaum J.S., Holford N.H.G., Sadée W. (1985). In Vivo Receptor Binding of Opioid Drugs at the μ Site. J. Pharmacol. Exp. Ther..

[68] Holze F., Vizeli P., Ley L., Müller F., Dolder P., Stocker M., Duthaler U., Varghese N., Eckert A., Borgwardt S. (2021). Acute dose-dependent effects of lysergic acid diethylamide in a double-blind placebo-controlled study in healthy subjects. Neuropsychopharm.

[69] Schmid Y., Enzler F., Gasser P., Grouzmann E., Preller K.H., Vollenweider F.X., Brenneisen R., Müller F., Borgwardt S., Liechti M.E. (2015). Acute effects of lysergic acid diethylamide in healthy subjects. Biol. Psychiatry.

[70] Dolder P.C., Schmid Y., Haschke M., Rentsch K.M., Liechti M.E. (2015). Pharmacokinetics and concentration-effect relationship of oral LSD in humans. Int. J. Neuropsychopharmacol..

[71] Dolder P.C., Schmid Y., Steuer A.E., Kraemer T., Rentsch K.M., Hammann F., Liechti M.E. (2017). Pharmacokinetics and pharmacodynamics of lysergic acid diethylamide in healthy subjects. Clin. Pharmacokinet..

[72] Leysen J.E., Janssen P.F., Niemegeers C.J. (1989). Rapid desensitization and down-regulation of 5-HT2 receptors by DOM treatment. Eur. J. Pharmacol..

[73] Kim K., Che T., Panova O., DiBerto J.F., Lyu J., Krumm B.E., Wacker D., Robertson M.J., Seven A.B., Nichols D.E. (2020). Structure of a hallucinogen-activated Gq-coupled 5-HT_2A_ serotonin receptor. Cell.

[74] Wacker D., Wang S., McCorvy J.D., Betz R.M., Venkatakrishnan A.J., Levit A., Lansu K., Schools Z.L., Che T., Nichols D.E. (2017). Crystal structure of an LSD-bound human serotonin receptor. Cell.

[75] Hartig P.R., Scheffel U., Frost J.J., Wagner H.N. (1985). In vivo binding of 125I-LSD to serotonin 5-HT2 receptors in mouse brain. Life Sci..

[76] Banks M.L., Hutsell B.A., Blough B.E., Poklis J.L., Negus S.S. (2015). Preclinical assessment of lisdexamfetamine as an agonist medication candidate for cocaine addiction: Effects in rhesus monkeys trained to discriminate cocaine or to self-administer cocaine in a cocaine versus food choice procedure. Int. J. Neuropsychopharmacol..

[77] Shang G.W., Liu D.N., Yan L.H., Cui X.Y., Zhang K.P., Qi C., Chen J. (2006). Nociceptive stimulus modality-related difference in pharmacokinetic-pharmacodynamic modeling of morphine in the rat. Pharmacol. Biochem. Behav..

[78] Albrecht S., Ihmsen H., Hering W., Geisslinger G., Dingemanse J., Schwilden H., Schüttler J. (1999). The effect of age on the pharmacokinetics and pharmacodynamics of midazolam. Clin. Pharmacol. Ther..

[79] Cocchetto D.M., Owens S.M., Perez-Reyes M., DiGuiseppi S., Miller L.L. (1981). Relationship between plasma delta-9-tetrahydrocannabinol concentration and pharmacologic effects in man. Psychopharmacology.

[80] Ren T., Zhu X., Jusko N.M., Krzyzanski W., Jusko W.J. (2022). Pharmacodynamic model of slow reversible binding and its applications in pharmacokinetic/pharmacodynamic modeling: Review and tutorial. J. Pharmacokinet. Pharmacodyn..

[81] Michel M.C., Michel-Reher M.B., Hein P. (2020). A systematic review of inverse agonism at adrenoceptor subtypes. Cells.

[82] Wang D., Raehal K.M., Bilsky E.J., Sadée W. (2001). Inverse agonists and neutral antagonists at μ opioid receptor (MOR): Possible role of basal receptor signaling in narcotic dependence. J. Neurochem..

[83] Piñeyro G., Azzi M., deLean A., Schiller P.W., Bouvier M. (2005). Reciprocal regulation of agonist and inverse agonist signaling efficacy upon short-term treatment of the human delta-opioid receptor with an inverse agonist. Mol. Pharmacol..

[84] Sato J., Makita N., Iiri T. (2016). Inverse agonism: The classic concept of GPCRs revisited. Endocr. J..

[85] Sirohi S., Dighe S.V., Madia P.A., Yoburn B.C. (2009). The relative potency of inverse opioid agonists and a neutral opioid antagonist in precipitated withdrawal and antagonism of analgesia and toxicity. J. Pharmacol. Exp. Ther..

[86] Ko M.C., Divin M.F., Lee H., Woods J.H., Traynor J.R. (2006). Differential in vivo potencies of naltrexone and 6β-naltrexol in the monkey. J. Pharmacol. Exp. Ther..

[87] Yancey-Wrona J., Dallaire B., Bilsky E.J., Bath B., Burkart J., Wenster L., Magiera D., Yang X., Phelps M.A., Sadee W. (2011). 6β-Naltrexol, a peripherally selective opioid antagonist that inhibits morphine-induced slowing of gastrointestinal transit: An exploratory study. Pain. Med..

[88] Porter S.J., Somogyi A.A., White J.M. (2002). In vivo and in vitro potency studies of 6beta-naltrexol, the major human metabolite of naltrexone. Addict. Biol..

[89] Raehal K.M., Lowery J.J., Bhamidipati C.M., Paolino R.M., Blair J.R., Wang D., Sadée W., Bilsky E.J. (2005). In vivo characterization of 6β-naltrexol, an opioid ligand with less inverse agonist activity compared with naltrexone and naloxone in opioid-dependent mice. J. Pharmacol. Exp. Ther..

[90] Wang Q., Dong X., Hu T., Qu C., Lu J., Zhou Y., Li J., Pei G. (2021). Constitutive activity of serotonin receptor 6 regulates human cerebral organoids formation and depression-like behaviors. Stem Cell Rep..

[91] Wang D., Sun X., Sadee W. (2011). 2007 Different effects of opioid antagonists on mu, delta, and kappa opioid receptors with and without agonist pretreatment. J. Pharmacol. Exp. Ther..

[92] Muneta-Arrate I., Diez-Alarcia R., Horrillo I., Meana J.J. (2020). Pimavanserin exhibits serotonin 5-HT2A receptor inverse agonism for Gαi1- and neutral antagonism for Gαq/11-proteins in human brain cortex. Eur. Neuropsychopharm..

[93] Chilcoat H.D., Amick H.R., Sherwood M.R., Dunn K.E. (2019). Buprenorphine in the United States: Motives for abuse, misuse, and diversion. J. Subst. Abuse Treat..

[94] Yokell M.A., Zaller N.D., Green T.C., Rich J.D. (2011). Buprenorphine and buprenorphine/naloxone diversion, misuse, and illicit use: An international review. Curr. Drug Abuse Rev..

[95] Cowan A., Lewis J.W., Macfarlane I.R. (1977). Agonist and antagonist properties of buprenorphine, a new antinociceptive agent. Br. J. Pharmaco..

[96] Colom M., Vidal B., Zimmer L. (2019). Is There a role for GPCR agonist radiotracers in PET neuroimaging?. Front. Mol. Neurosc..

[97] Mangeant R., Dubost E., Cailly T., Collot V. (2022). Radiotracers for the central serotoninergic system. Pharmaceuticals.

[98] Sleight A.J., Stam N., Mutel V., Vanderheyden P.M. (1996). Radiolabelling of the human 5-HT2A receptor with an agonist, a partial agonist and an antagonist: Effects on apparent agonist affinities. Biochem. Pharmacol..

[99] Crilly S.E., Ko W., Weinberg Z.Y., Puthenveedu M.A. (2021). Conformational specificity of opioid receptors is determined by subcellular location irrespective of agonist. Elife.

[100] Radoux-Mergault A., Oberhauser L., Aureli S., Gervasio F.L., Stoeber M. (2023). Subcellular location defines GPCR signal transduction. Sci. Adv..

[101] Cao C., Barros-Álvarez X., Zhang S., Kim K., Dämgen M.A., Panova O., Suomivuori C.M., Fay J.F., Zhong X., Krumm B.E. (2022). Signaling snapshots of a serotonin receptor activated by the prototypical psychedelic LSD. Neuron.

[102] Vargas M.V., Dunlap L.E., Dong C., Carter S.J., Tombari R.J., Jami S.A., Cameron L.P., Patel S.D., Hennessey J.J., Saeger H.N. (2023). Psychedelics promote neuroplasticity through the activation of intracellular 5-HT2A receptors. Science.

[103] Corder G., Doolen S., Donahue R.R., Winter M.K., Jutras B.K.L., He Y., Hu X., Wieskopf J.S., Mogil J.S., Storm D.R. (2013). Constitutive μ-opioid receptor activity leads to long-term endogenous analgesia and dependence. Science.

[104] Navani D.M., Sirohi S., Madia P.A., Yoburn B.C. (2011). The role of opioid antagonist efficacy and constitutive opioid receptor activity in the opioid withdrawal syndrome in mice. Pharm. Biochem. Behav..

[105] Shoblock J.R., Maidment N.T. (2007). Enkephalin release promotes homeostatic increases in constitutively active mu opioid receptors during morphine withdrawal. Neuroscience.

[106] Walwyn W., Evans C.J., Hales T.G. (2007). Beta-arrestin2 and c-Src regulate the constitutive activity and recycling of mu opioid receptors in dorsal root ganglion neurons. J. Neurosci..

[107] Liu J.G., Prather P.L. (2001). Chronic exposure to mu-opioid agonists produces constitutive activation of mu-opioid receptors in direct proportion to the efficacy of the agonist used for pretreatment. Mol. Pharmacol..

[108] Liu J.G., Prather P.L. (2002). Chronic agonist treatment converts antagonists into inverse agonists at delta-opioid receptors. J. Pharmacol. Exp. Ther..

[109] Belcheva M.M., Szùcs M., Wang D., Sadee W., Coscia C.J. (2001). μ-Opioid receptor-mediated ERK activation involves calmodulin-dependent epidermal growth factor receptor transactivation. J. Biol. Chem..

[110] Safa A., Lau A.R., Aten S., Schilling K., Bales K.L., Miller V., Fitzgerald J., Chen M., Hill K., Dzwigalski K. (2021). Pharmacological prevention of neonatal opioid withdrawal in a pregnant guinea pig model. Front. Pharmacol..

[111] Oberdick J., Ling Y., Phelps M.A., Yudovich M.S., Schilling K., Sadee W. (2016). Preferential delivery of an opioid antagonist to the fetal brain in pregnant mice. J. Pharmacol. Exp. Ther..

[112] Sullivan L.C., Chavera T.S., Jamshidi R.J., Berg K.A., Clarke W.P. (2016). Constitutive desensitization of opioid receptors in peripheral sensory neurons. J. Pharmacol. Exp. Ther..

[113] Borroto-Escuela D.O., Romero-Fernandez W., Narvaez M., Oflijan J., Agnati L.F., Fuxe K. (2014). Hallucinogenic 5-HT2AR agonists LSD and DOI enhance dopamine D2R protomer recognition and signaling of D2-5-HT2A heteroreceptor complexes. Biochem. Biophys. Res. Commun..

[114] Bockaert J., Bécamel C., Chaumont-Dubel S., Claeysen S., Vandermoere F., Marin P. (2021). Novel and atypical pathways for serotonin signaling. Fac. Rev..

[115] Toneatti R., Shin J.M., Shah U.H., Mayer C.R., Saunders J.M., Fribourg M., Arsenovic P.T., Janssen W.G., Sealfon S., López-Giménez J.F. (2020). Interclass GPCR heteromerization affects localization and trafficking. Sci. Signal..

[116] Berg K.A., Harvey J.A., Spampinato U., Clarke W.P. (2008). Physiological and therapeutic relevance of constitutive activity of 5-HT 2A and 5-HT 2C receptors for the treatment of depression. Prog. Brain Res..

[117] Aloyo V.J., Berg K.A., Clarke W.P., Spampinato U., Harvey J.A. (2010). Inverse agonism at serotonin and cannabinoid receptors. Prog. Mol. Biol. Transl. Sci..

[118] Kantrowitz J.T. (2020). Targeting serotonin 5-HT_2A_ receptors to better treat schizophrenia: Rationale and current approaches. CNS Drugs.

[119] López-Giménez J.F., González-Maeso J. (2018). Hallucinogens and serotonin 5-HT_2A_ receptor-mediated signaling pathways. Curr. Top. Behav. Neurosci..

[120] Rickli A., Moning O.D., Hoener M.C., Liechti M.E. (2016). Receptor interaction profiles of novel psychoactive tryptamines compared with classic hallucinogens. Eur. Neuropsychopharmacol..

[121] McGuire A.L., Lynch H.F., Grossman L.A., Cohen I.G. (2023). Pressing regulatory challenges for psychedelic medicine. Science.

[122] Schindler E.A.D., D’Souza D.C. (2022). The therapeutic potential of psychedelics. Science.

[123] Slocum S.T., DiBerto J.F., Roth B.L. (2022). Molecular insights into psychedelic drug action. J. Neurochem..

[124] Galvão-Coelho N.L., Marx W., Gonzalez M., Sinclair J., de Manincor M., Perkins D., Sarris J. (2021). Classic serotonergic psychedelics for mood and depressive symptoms: A meta-analysis of mood disorder patients and healthy participants. Psychopharmacology.

[125] Ly C., Greb A., Vargas M.V., Duim W.C., Grodzki A.C.G., Lein P.J., Olson D.E. (2020). Transient stimulation with psychoplastogens is sufficient to initiate neuronal growth. ACS Pharmacol. Transl. Sci..

[126] Kolaczynska K.E., Luethi D., Trachsel D., Hoener M.C., Liechti M.E. (2022). Receptor interaction profiles of 4-alkoxy-3,5-dimethoxy-phenethylamines (mescaline derivatives) and related amphetamines. Front. Pharmacol..

[127] Barker S.A., McIlhenny E.H., Strassman R. (2012). A critical review of reports of endogenous psychedelic N, N-dimethyltryptamines in humans: 1955–2010. Drug Test. Anal..

[128] Dean J.G., Liu T., Huff S., Sheler B., Barker S.A., Strassman R.J., Wang M.M., Borjigin J. (2019). Biosynthesis and extracellular concentrations of N,N-dimethyltryptamine (DMT) in mammalian brain. Sci. Rep..

[129] Roth B.L., Gumpper R.H. (2023). Psychedelics as transformative therapeutics. Am. J. Psychiatry.

[130] Family N., Hendricks P.S., Williams L.T., Luke D., Krediet E., Maillet E.L., Raz S. (2022). Safety, tolerability, pharmacokinetics, and subjective effects of 50, 75, and 100 µg LSD in healthy participants within a novel intervention paradigm: A proof-of-concept study. J. Psychopharmacol..

[131] Shao L.X., Liao C., Gregg I., Davoudian P.A., Savalia N.K., Delagarza K., Kwan A.C. (2021). Psilocybin induces rapid and persistent growth of dendritic spines in frontal cortex in vivo. Neuron.

[132] Seifert R., Wenzel-Seifert K. (2002). Constitutive activity of G-protein-coupled receptors: Cause of disease and common property of wild-type receptors. Naunyn Schmiedebergs Arch. Pharmacol..

[133] Moliner R., Girych M., Brunello C.A., Kovaleva V., Biojone C., Enkavi G., Antenucci L., Kot E.F., Goncharuk S.A., Kaurinkoski K. (2023). Psychedelics promote plasticity by directly binding to BDNF receptor TrkB. Nat. Neurosci..

[134] Casarotto P.C., Girych M., Fred S.M., Kovaleva V., Moliner R., Enkavi G., Biojone C., Cannarozzo C., Sahu M.P., Kaurinkoski K. (2021). Antidepressant drugs act by directly binding to TRKB neurotrophin receptors. Cell.

[135] Rantamäki T., Hendolin P., Kankaanpää A., Mijatovic J., Piepponen P., Domenici E., Chao M.V., Männistö P.T., Castrén E. (2007). Pharmacologically diverse antidepressants rapidly activate brain-derived neurotrophic factor receptor TrkB and induce phospholipase-Cgamma signaling pathways in mouse brain. Neuropsychopharmacology.

[136] Barker S.A. (2022). Administration of N,N-dimethyltryptamine (DMT) in psychedelic therapeutics and research and the study of endogenous DMT. Psychopharmacology.

[137] Polito V., Liknaitzky P. (2022). The emerging science of microdosing: A systematic review of research on low dose psychedelics (1955-2021) and recommendations for the field. Neurosci. Biobehav. Rev..

[138] Yin Y., Li Y., Zhang W. (2014). The growth hormone secretagogue receptor: Its intracellular signaling and regulation. Int. J. Mol. Sci..

[139] Davis J. (2018). Hunger, ghrelin and the gut. Brain Res..

[140] Gupta S., Mukhopadhyay S., Mitra A. (2022). Therapeutic potential of GHSR-1A antagonism in alcohol dependence, a review. Life Sci..

[141] Cornejo M.P., Mustafá E.R., Barrile F., Cassano D., De Francesco P.N., Raingo J., Perello M. (2021). The intriguing ligand-dependent and -independent actions of the growth hormone secretagogue receptor on reward related behaviors. Neurosci. Biobehav. Rev..

[142] Mear Y., Enjalbert A., Thirion S. (2013). GHS-R1a constitutive activity and its physiological relevance. Front. Neurosci..

[143] Pantel J., Legendre M., Cabrol S., Hilal L., Hajaji Y., Morisset S., Nivot S., Vie-Luton M.-P., Grouselle D., de Kerdanet M. (2006). Loss of constitutive activity of the growth hormone secretagogue receptor in familial short stature. J. Clin. Investig..

[144] Torz L.J., Osborne-Lawrence S., Rodriguez J., He Z., Cornejo M.P., Mustafá E.R., Jin C., Petersen N., Hedegaard M.A., Nybo M. (2020). Metabolic insights from a GHSR-A203E mutant mouse model. Mol. Metab..

[145] Cornejo M.P., Castrogiovanni D., Schiöth H.B., Reynaldo M., Marie J., Fehrentz J.A., Perello M. (2019). Growth hormone secretagogue receptor signalling affects high-fat intake independently of plasma levels of ghrelin and LEAP2, in a 4-day binge eating model. J. Neuroendocrinol..

[146] Ibrahim Abdalla M.M. (2015). Ghrelin—Physiological functions and regulation. Eur. Endocrinol..

[147] Kazius J., Wurdinger K., Van Iterson M., Kok J., Bäck T., Ijzerman A.P. (2008). GPCR NaVa database: Natural variants in human G protein-coupled receptors. Hum. Mutat..

[148] Pándy-Szekeres W., Caroli J., Marmybekov A., Kermani A.A., Kserü G.M., Kooistra A.J., Gloriam D.E. (2023). GPCRdb in 2023: State-specific structure models using AlphaFold2 and new ligand resources. Nucleic Acids Res..

[149] Chen G., Way J., Armour S., Watson C., Queen K., Jayawickreme C.K., Chen W.J., Kenakin T. (2000). Use of constitutive G protein-coupled receptor activity for drug discovery. Mol. Pharmacol..

[150] Luzum J.A., Sweet K.M., Binkley P.F., Schmidlen T.J., Jarvis J.P., Christman M.F., Sadee W., Kitzmiller J.P. (2017). CYP2D6 genetic variation and beta-blocker maintenance dose in patients with heart failure. Pharm. Res..

[151] Hainer V., Aldhoon Hainerová I., Kunešová M., Taxová Braunerová R., Zamrazilová H., Bendlová B. (2020). Melanocortin pathways: Suppressed and stimulated melanocortin-4 receptor (MC4R). Physiol. Res..

[152] Kovacs P., Schöneberg T. (2016). The relevance of genomic signatures at adhesion GPCR loci in humans. Handb. Exp. Pharmacol..

[153] Unal H., Karnik S.S. (2014). Constitutive activity in the angiotensin II type 1 receptor: Discovery and applications. Adv. Pharmacol..

[154] Xu B., Chakraborty R., Eilers M., Dakshinamurti S., O’Neil J.D., Smith S.O., Bhullar R.P., Chelikani P. (2013). High-level expression, purification and characterization of a constitutively active thromboxane A2 receptor polymorphic variant. PLoS ONE.

[155] Ward R.J., Pediani J.D., Marsango S., Jolly R., Stoneman M.R., Biener G., Handel T.M., Raicu V., Milligan G.J. (2021). Chemokine receptor CXCR4 oligomerization is disrupted selectively by the antagonist ligand IT1t. Biol. Chem..

[156] Vassart G., Costagliola S. (2011). G protein-coupled receptors: Mutations and endocrine diseases. Nat. Rev. Endocrinol..

[157] Helfinger L., Tate C.G. (2022). Expression and purification of the human thyroid-stimulating hormone receptor. Methods Mol. Biol..

[158] Athanasiou D., Aguila M., Bellingham J., Li W., McCulley C., Reeves P.J., Cheetham M.E. (2018). The molecular and cellular basis of rhodopsin retinitis pigmentosa reveals potential strategies for therapy. Prog. Retin. Eye Res..

[159] Patchett A.A., Nargund R.P., Tata J.R., Chen M.H., Barakat K.J., Johnston D.B., Cheng K., Chan W.W., Butler B., Hickey G. (1995). Design and biological activities of L-163,191 (MK-0677): A potent, orally active growth hormone secretagogue. Proc. Natl. Acad. Sci. USA.

[160] Arang N., Gutkind J.S. (2020). G Protein-Coupled receptors and heterotrimeric G proteins as cancer drivers. FEBS Lett..

[161] Chaudhary P.K., Kim S. (2021). An insight into GPCR and G-proteins as cancer drivers. Cells.

[162] Spiegelberg B.D., Hamm H.E. (2007). Roles of G-protein-coupled receptor signaling in cancer biology and gene transcription. Curr. Opin. Genet. Dev..

[163] Bongers B.J., Gorostiola González M., Wang X., van Vlijmen H.W.T., Jespers W., Gutiérrez-de-Terán H., Ye K., IJzerman A.P., Heitman L.H., van Westen G.J.P. (2022). Pan-cancer functional analysis of somatic mutations in G protein-coupled receptors. Sci. Rep..

[164] Sriram K., Moyung K., Corriden R., Carter H., Insel P.A. (2019). GPCRs show widespread differential mRNA expression and frequent mutation and copy number variation in solid tumors. PLoS Biol..

[165] Li S., Chen X., Chen J., Wu B., Liu J., Guo Y., Li M., Pu X. (2023). Multi-omics integration analysis of GPCRs in pan-cancer to uncover inter-omics relationships and potential driver genes. Comput. Biol. Med..

[166] Dorsam R.T., Gutkind J.S. (2007). G-protein-coupled receptors and cancer. Nat. Rev. Cancer.

[167] Li S., Huang S., Peng S.B. (2005). Overexpression of G protein-coupled receptors in cancer cells: Involvement in tumor progression. Int. J. Oncol..

[168] Nugent A., Proia R.L. (2017). The role of G protein-coupled receptors in lymphoid malignancies. Cell Signal..

[169] Wang X., Jespers W., de Waal J.J., Wolff K.A.N., Van Uden L., IJzerman A.P., van Westen G.J.P., Heitman L.H. (2022). Cancer-related somatic mutations alter adenosine A_1_ receptor pharmacology—A focus on mutations in the loops and C-terminus. FASEB J..

[170] Jiang J. (2022). Hedgehog signaling mechanism and role in cancer. Semin. Cancer Biol..

[171] Zhang H., Sun Z., Liu Z., Song C. (2018). Overcoming the emerging drug resistance of smoothened: An overview of small-molecule SMO antagonists with antiresistance activity. Future Med. Chem..

[172] Raymond J.H., Aktary Z., Larue L., Delmas V. (2022). Targeting GPCRs and their signaling as a therapeutic option in melanoma. Cancers.

